# Highly efficient mode-locked and Q-switched Er^3+^-doped fiber lasers using a gold nanorod saturable absorber

**DOI:** 10.1038/s41598-021-99676-0

**Published:** 2021-10-11

**Authors:** Yin-Wen Lee, Chien-Ming Chen, Wei-Hsiang Chuang, Ching-Yi Cho, Cheng-Hsien Yu, M. C. Paul

**Affiliations:** 1 Department of Electro-Optical Engineering, National Taipei University of Technology, 1, Sec. 3, Zhongxiao E. Rd., Taipei, 10608 Taiwan; 2 CSIR-Central Glass and Ceramic Research Institute, 196 Raja S. C. Mullick Road, Kolkata, 700032 West Bengal India

**Keywords:** Nanoscience and technology, Optics and photonics

## Abstract

Mode-locked and Q-switched pulsed fiber laser sources with wavelengths of 1.55 μm are widely used in various fields. Gold nanorods (GNRs) have been applied in biomedicine and optics owing to their biocompatibility, easy fabrication, and unique optical properties. This paper presents the analysis of a saturable absorber based on a colloidal gold nanorod (GNR) thin film for dual-function passively mode-locked and Q-switched 1.55-μm fiber lasers. The colloidal GNR thin film possesses superior properties such as a wide operating wavelength range, large nonlinear absorption coefficient, and a picosecond-order recovery time. Its modulation depth and saturation intensity at 1.55 μm are 7.8% and 6.55 MW/cm^2^, respectively. Passive mode-locked or Q-switched laser operation is achieved by changing the number of GNR thin-film layers. The advantages of these high-quality GNRs in mode-locked and Q-switched fiber lasers with record-high slope efficiency are verified by conducting comprehensive material and laser dynamic analyses. The self-starting mode-locked fiber laser with an efficiency as high as 24.91% and passively Q-switched fiber laser with the maximum energy of 0.403 μJ are successfully demonstrated. This paper presents the novel demonstration of reconfigurable mode-locked and Q-switched all-fiber lasers by incorporating colloidal GNR thin films.

## Introduction

Mode-locked and Q-switched pulsed fiber laser sources operating at 1.55 μm have attracted widespread interest owing to their practical applications in optical communication, LIDA, and medical surgery. They have been fabricated in various formats, with pulse durations ranging from femtoseconds to a few nanoseconds, and they offer several advantages over their free-space counterparts, including compactness, greater mechanical stability, and ease of maintenance. Both active and passive switching elements are typically employed in pulsed fiber laser systems. Although active mode-locking or Q-switching of fiber lasers enables stable pulsed operation, this generally requires complex drive electronics or expensive active optical components^1^. Passively pulsed fiber laser systems can be fabricated in a simple all-fiber configuration, which is more cost-effective, by making use of saturable absorbers (SAs). Several types of materials have been fabricated for use as SAs in 1.55-μm pulsed fiber laser systems. These include semiconductor saturable absorber mirrors^2^, carbon nanotubes^3^, graphene^4^, topological insulators^5^, black phosphorus^6^, and quantum dots^7^. The requirement for broadband nonlinear optical responses as well as efficient and cost-effective devices has prompted extensive research into novel SA materials.

Considerable attention has been paid to gold nanorods (GNRs) in the past decade because their biocompatibility, easy fabrication, and unique optical properties makes them highly applicable to various fields in biomedicine and optics^8,9^. They possess great potential as SAs in pulsed fiber lasers owing to their picosecond-order recovery time^10^, broadband surface plasmon resonance (SPR) absorption^11^, and large third-order nonlinear coefficients^12^. GNRs have two SPR absorption bands, unlike gold nanoparticles, which have one, because of their anisotropic nature^12^. The first is the transverse SPR absorption band, which is vertical to the axial of the pole^13^. The other is the longitudinal SPR absorption band, which is generated along the axial direction of the pole and varies based on the nanorod aspect ratio and overall size^9,10^. The different absorption mechanisms can broaden the operating wavelength range of GNRs, making them a promising broadband nonlinear optical material. In 2013, the first GNR-based mode-locked and Q-switched fiber lasers were reported^14,15^. GNR SAs have since been employed in Yb^3+^, Er^3+^, and Tm^3+^-doped pulsed fiber lasers^15–17^. In addition, GNR SAs have been implemented in various platforms such as microfibers^18^, fiber ferrules^19^, side-polished fibers^20^, and colloidal thin films^14^. Most of these platforms generate either Q-switched or mode-locked pulses. Only one dual-function fiber laser, which offers mode-locked pulses and multi-wavelength Q-switched pulses, with switching between these modes by polarization adjustment, has been presented^21^. However, to the best of our knowledge, all the previously reported GNR-based fiber laser systems have relatively low slope efficiencies, and numerical simulations have not been thoroughly analyzed to support the experimental results. Therefore, this study presents the fabrication process and material properties of GNR thin-film SAs, along with the demonstration of efficient mode-locked and Q-switched Er^3+^-doped YAS fiber lasers. Furthermore, it also provides the numerical and simulation analyses of efficient narrow-band soliton mode-locked and Q-switched fiber lasers.

## Methods

### Gold nanorod thin film fabrication and optical characteristics

**Figure 1:**
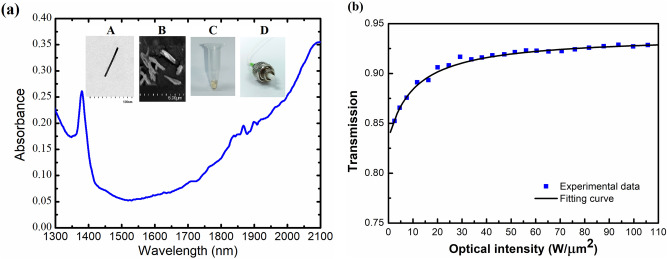
Gold nanorod (GNR) thin film characterization. (**a**) Absorption spectrum of the GNR thin film. The insets show A: TEM image, B: SEM image, C: Photograph of the GNR water solution, and D: Photograph of the fiber compatible GNR-SA film. (**b**) Saturable absorption characteristics of GNR QD thin film at 1565 nm.

The GNR thin film exhibits a better potential for use within an all-fiber laser system, sandwiched between two fiber connectors, as compared to other device geometries. The GNRs used in this study were purchased from Creative Diagnostics. They were cetyltrimethylammonium bromide (CTAB) capped and dissolved in DI water. The transmission electron microscopy (TEM) image in the inset of Fig. 1 shows the gold nanorod with an average diameter and length of 10 nm and 175 nm, respectively^22^. The coefficient of variation in GNR size distribution is less than 10%^22^. In general, the geometry of metal nanoparticles significantly affects the SPR interaction between the laser and the particles. The water-dissolved GNRs are specified by the manufacturer to have a maximum longitudinal SPR absorbance at 2100 nm. The GNR polymer thin film was fabricated as follows: GNR solution (200 μL) was first centrifuged at 12,000 rpm for 15 min, as shown in the inset of Fig. 1. After removing 150 μL of the supernatant, the remaining 50 μL of the condensed GNR solution was mixed with 50 µL of aqueous polyvinyl alcohol (PVA) solution. Subsequently, 4 µL of the dispersant was added to 100 μL of GNR within the PVA solution to ensure the uniform distribution of the GNRs. The scanning electron microscope (SEM) image in the inset of Fig. 1 indicates the GNR doped in the films. A One-Touch Vortexer Mixer (FINEPCR) was used to uniformly distribute the GNRs throughout the PVA polymer and then the GNR-PVA was dip-coated onto a glass slide. The plated film was first stored in a refrigerator at 4 °C for three days and then at room temperature for two days to maintain the chemical stability of the film. The film was torn off the slide and cut into appropriate sizes during usage. The thickness of the completed GNR-PVA film was measured with a coating thickness gauge (Elcometer Co., UK), and an average thickness of 31.2 µm was obtained.

In addition to the GNR geometry, environmental conditions also result in different nanorod responses. Figure 1a presents the absorbance spectrum of the 31.2-μm film which is measured using a spectrometer (Agilentcary5000). The surface plasmon resonance is divided into longitudinal and lateral resonances as the GNRs have an anisotropic structure. A maximum absorption peak can be observed at a wavelength of 2100 nm, which corresponds to the longitudinal surface plasmon resonance. A second absorption peak at 1380 nm is assigned to the transverse surface plasmon resonance. Typically, the modulation depth, non-saturable absorber, recovery time, and saturation intensity are essential parameters that affect the output performance of mode-locked pulsed lasers. Therefore, these parameters of the GNR thin film were characterized by measuring its transmission as a function of incident peak power. A homemade mode-locked all-fiber system based on an erbium-doped fiber (EDF) laser was used as the light source. The laser has the maximum pulse energy of 3.2 nJ with a pulse width of 644 fs. The details of the system configuration and measurement method are presented in Sect. 2 of Ref.^7^. Figure 1b shows the measured transmittance as a function of the incident intensity. The data were fitted using a two-level saturable absorption model^7^. The fitted curve shows a modulation depth of 7.8% and a saturation intensity of 6.55 MW/cm^2^.

### Experimental setup

**Figure 2:**
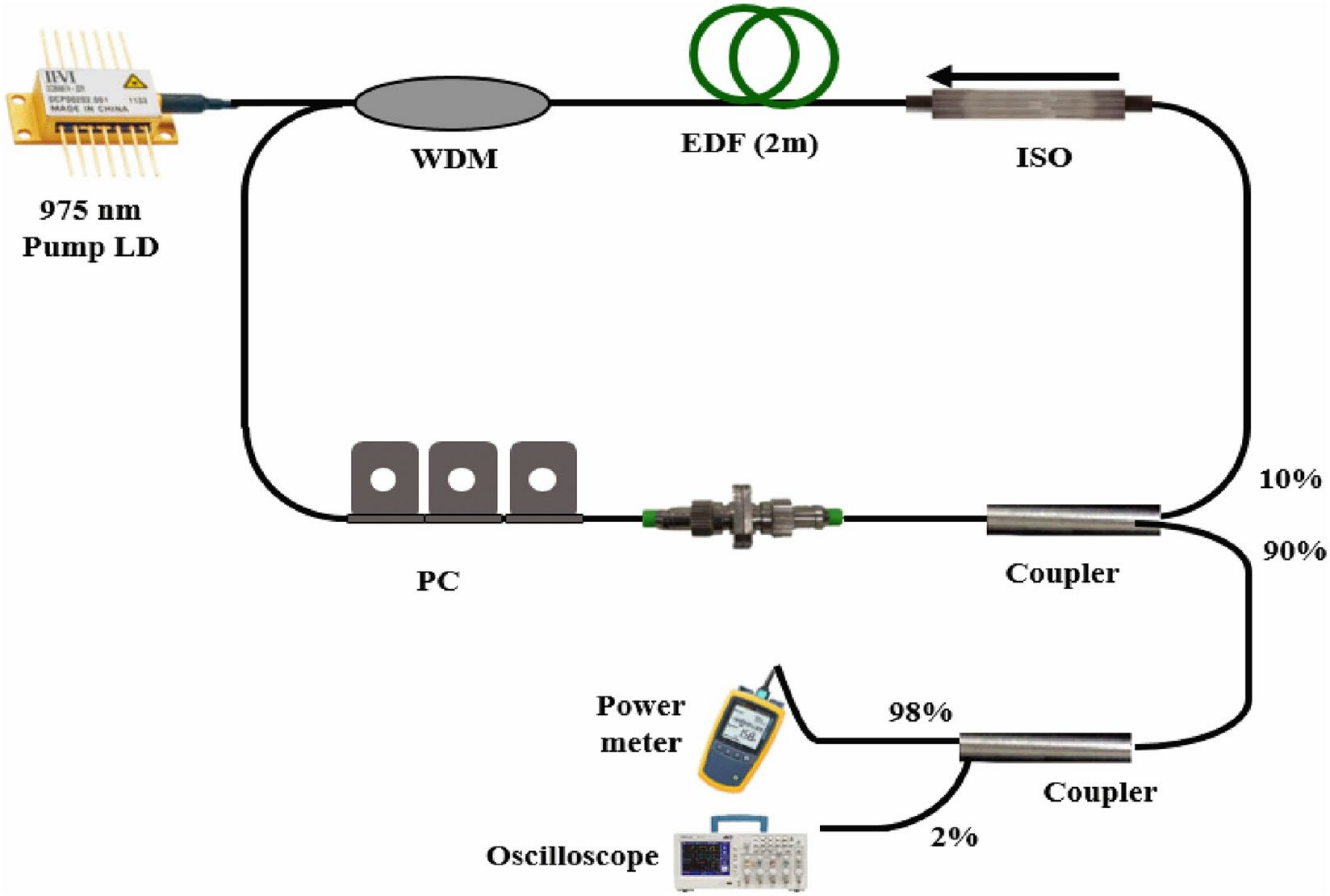
Experimental setup of the gold nanorod saturable absorber (GNR-SA)-based Er^3+^-doped yttria-stabilized zirconia aluminosilicate (YSZA) pulsed fiber laser.

This study successfully demonstrated both mode-locked and Q-switched laser operation within the same laser configuration by using different numbers of GNR film layers. Figure 2 shows the experimental setup of the 975-nm counter-pumped 1.56-μm fiber ring laser. An Er^3+^-doped nano-engineered yttria-stabilized zirconia aluminosilicate (YSZA) fiber was employed as the gain medium. This fiber allows for efficient laser emission because of the elimination of Er^3+^-ion cluster formation^23^. It has a core diameter of 10 μm and an NA of 0.2. The upper state lifetime and background loss are 10.47 ms and 50–110 dB/km, respectively. The ring laser cavity had a total cavity length of 17.7 m, consisting of a 15.5-m standard SMF-28 fiber and a 2-m Er^3+^-doped YSZA fiber, which was chosen to achieve more than 95% pump absorption. A polarization-dependent isolator was installed to ensure unidirectional laser propagation and a polarization controller was adopted only for the mode-locked laser to optimize the polarization state of the intracavity light. The GNR-SA films were sandwiched between two FC/APC connectors and then installed in the fiber ring cavity. Most of the intracavity laser intensity (90%) was coupled out of the ring via a 90/10 directional beam coupler. All of the output fiber ends were angle cleaved to avoid in-cavity back reflection, which can produce unstable mode-locking and Q-switching. The output laser average power was recorded using a power meter (Ophir 3A). The pulse time traces were monitored using a high-speed detector (EOT 3010) connected to an oscilloscope (Tektronix DPO3054) and a radio frequency (RF) spectrum analyzer (Agilent 8595E). An optical spectrum analyzer (HP 70952 B) and an autocorrelator (Femtochrome FR-103HP) were employed to measure the output laser spectrum and pulse duration.

## Results and discussion

### Mode-locked Er^3+^-doped YSZA fiber laser

**Figure 3:**
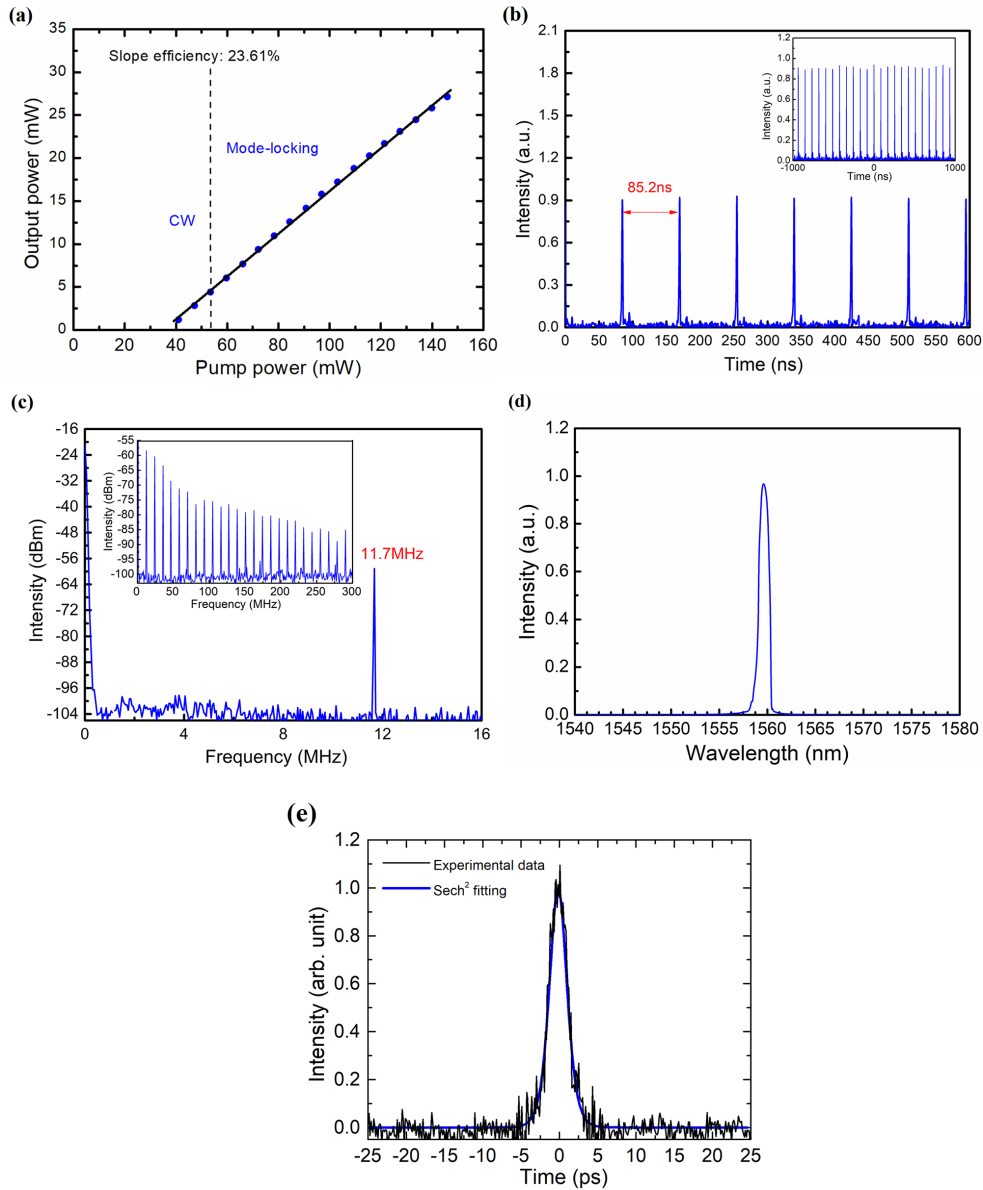
Mode-locked Er^3+^-doped yttria-stabilized zirconia aluminosilicate (YSZA) fiber laser operation. (**a**) Average output power versus pump power (blue dot) and linear fit to the data (black line). (**b**) Time trace, (**c**) Radio-frequency (RF) spectrum, (**d**) Laser output spectrum, and (**e**) Measured (black line) and hyperbolic-secant-fitted (blue line) auto-correlation curves.

A single-layer GNR-SA film was employed to achieve mode-locked laser operation. Figure 3 summarizes the mode-locked pulse parameters under the maximum output power. The EDF laser began CW lasing under a pump power of 41 mW. The generation of the CW mode-locking pulses was stabilized when the pump power reached 53.6 mW by appropriate adjustment of the polarization controller. This CW mode-locking threshold power is higher than that of the reported mode-locked Er^3+^-doped yttrium aluminosilicate (YAS) fiber laser using the nonlinear polarization rotation (NPR) mechanism^23^. Figure 3a shows the output laser average power as a function of the pump power. The maximum output power of 27.13 mW was limited by the unstable multi-pulse mode-locked operation. Although the relatively low reflectivity (10%) results in a higher pump threshold, it also produces a higher laser slope efficiency. The obtained 24.91% laser slope efficiency is comparable with that of the NPR mode-locked Er^3+^-doped YAS fiber lasers and is much higher than those of passive mode-locked Er^3+^-doped fiber lasers based on CNTs^3^ or graphene^4^. Figure 3b shows the output pulse train at the maximum laser power, for which the pulse interval is 85.2 ns, corresponding to a repetition rate of 11.7 MHz and confirming the cavity length of 17.7 m. The pulse intervals of different laser output powers are all measured as 85.2 ns that confirms the mode-locked operation. Figure 3c shows the measured RF spectrum with a frequency span of 300 MHz. Stable CW mode-locked operation was confirmed by a signal-to-noise ratio of 41.5 dB and a uniform harmonic spectrum without spectral modulation. Figure 3d shows the laser output spectrum which has a center wavelength of 1559.9 nm and a 3-dB bandwidth of 1.25 nm. The laser wavelength was determined through the automatic balance of gain competition, dispersion, and nonlinear effects inside the cavity. Figure 3e shows the autocorrelation trace which shows a 3.4-ps soliton pulse that can be successfully fitted with a sech^2^ curve. The maximum pulse energy and peak power were calculated as 2.34 nJ and 0.8 kW, respectively. The time-bandwidth product (TBP) is 0.53, which indicates that the pulse is slightly chirped.

**Figure 4:**
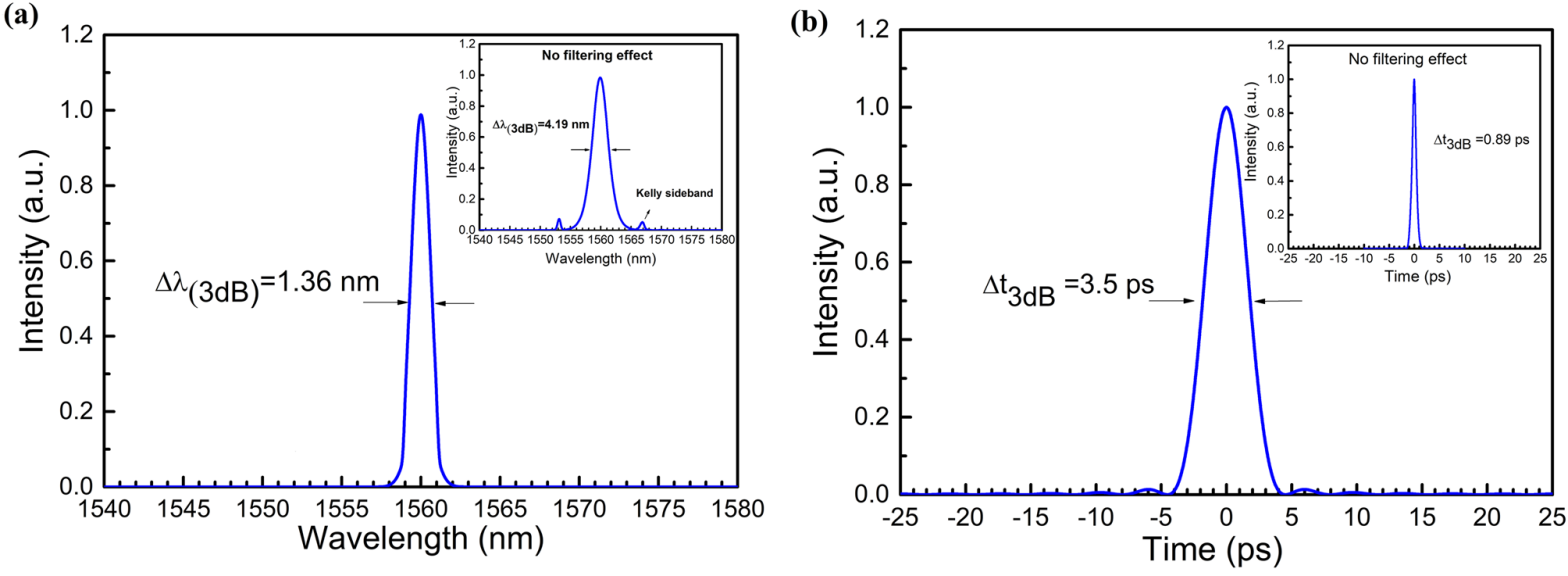
Mode-locked laser simulation results. (**a**) Laser spectrum and (**b**) Temporal profile. The insets show the simulation results of the same mode-locked fiber laser without the filtering effect.

At 1.55 μm, the group velocity dispersions for the Er^3+^-doped YAS and SMF28 fibers are − 29.3 ps^2^/km and − 22 ps^2^/km, respectively. The net cavity dispersion is thus estimated to be − 0.40 ps^2^. Such an anomalous net cavity dispersion usually results in the generation of conventional soliton pulses with Kelly sidebands^24^. However, the obtained laser pulses have no Kelly sidebands and have a narrow linewidth. The suppression of the Kelly sidebands is attributed to an additional filtering effect inside the laser cavity. Based on the results of Dai et al*.*, the added narrow-band filter eliminates the dispersive wave and thus suppresses the Kelly sidebands^25^. Similar to the filtering effect reported for NPR Er^3+^-doped mode-locked fiber lasers^26^, the highly birefringent GNR thin film combined with the PC and polarization-dependent isolator to form an intrinsic filter inside the cavity. It has been numerically and experimentally verified that the pulse duration increases and the spectral linewidth reduces as the filter bandwidth decreases^25^. In this study, numerical simulations were also performed using the commercial simulation tool Fiberdesk to analyze the narrow-band pulse generation and further understand the mode-locking dynamics. The abovementioned fiber structure and dispersion parameters were employed. As the results shown in Fig. 4a,b, the simulated 3-dB pulse duration, peak power, and spectral FWHM with the 2-nm filter successfully reflect the experimental observations. For the comparison, the numerical simulations for the same mode-locked Er^3+^-doped YAS fiber laser without the 2-nm filter were presented in the insets of Fig. 4a,b. These results confirm the mechanism of the intrinsic intra-cavity filter in our study.

### Q-switched Er^3+^-doped YSZA fiber laser

**Figure 5:**
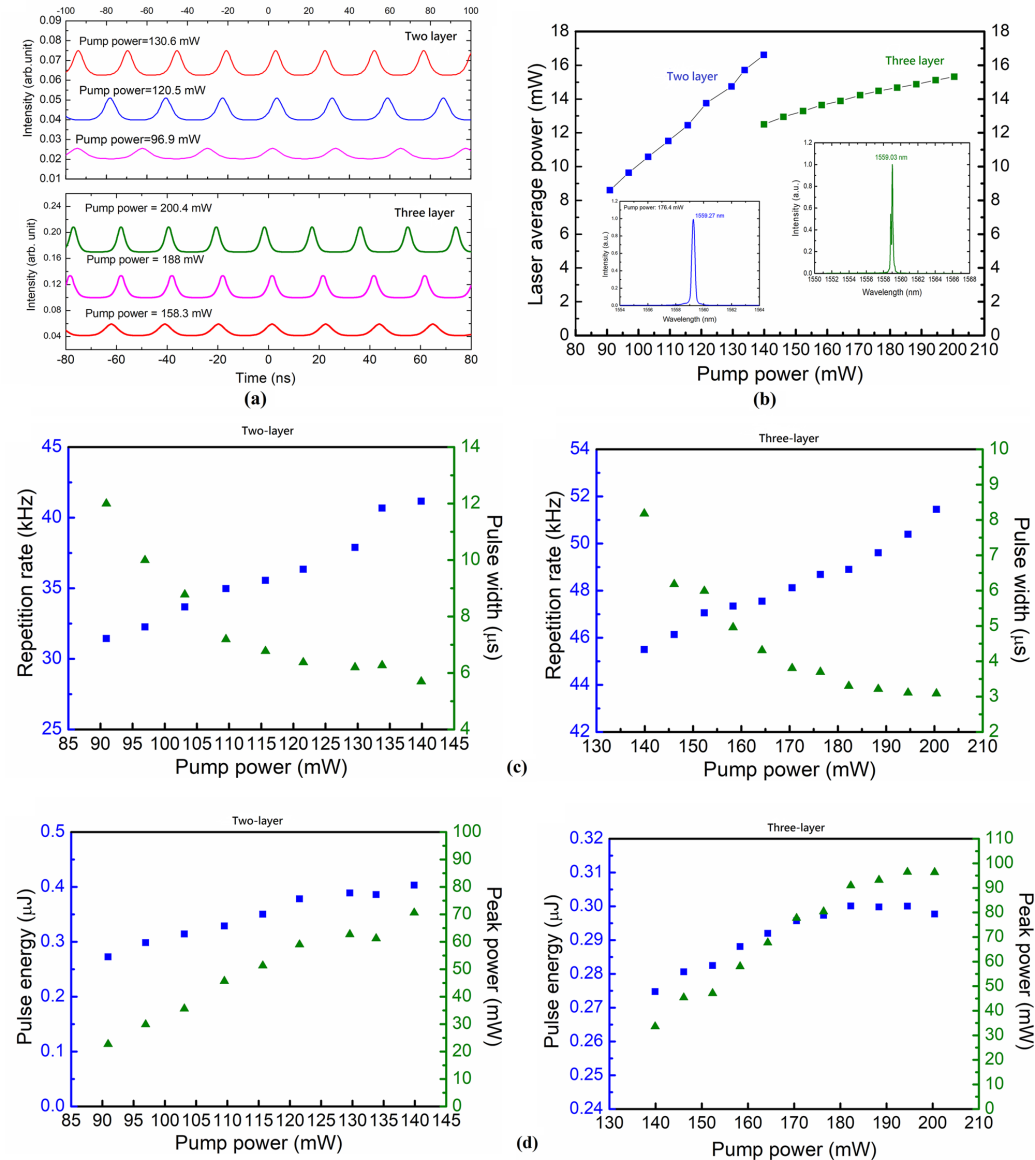
Q-switched Er^3+^-doped yttria-stabilized zirconia aluminosilicate (YSZA) fiber laser operation with two- and three-layer gold nanorod thin film saturable absorber (SA). (**a**) Oscilloscope traces of Q-switched pulse trains under different pump powers. (**b**) Measured average output power as a function of pump power. (**c**) Pulse duration and repetition rate (upper plots) and pulse energy and peak power (lower plots) versus pump power for the laser incorporating the two-layer SA. (**d**) Pulse energy and peak power (upper plots) and pulse energy and peak power (lower plots) as a function of pump power for the laser incorporating the three-layer SA.

The transition from mode-locked to Q-switched operation can be realized using multilayer GNR thin films. For the Q-switched laser operation, the same laser configuration is adopted as that shown in Fig. 2, except that a multi-layer GNR-SA was used in place of a single-layer GNR-SA and the PC was removed. Figure 5a depicts the oscilloscope trace of the Q-switched laser pulse trains generated with two and three layers of GNR-SA thin films under varying pump powers. The Q-switched pulses are relatively symmetric, and there is no sign of modulation due to mode-locking instability. The pulse interval decreases with increasing pump power, verifying that stable passively Q-switched (PQS) operation was realized. Figure 5b presents the average output power of the PQS lasers as a function of the launched pump power. The Q-switching threshold pump powers are 90.9 mW and 139.9 mW. Slope efficiencies of 16.37% and 4.55% were measured. Thanks to the use of an efficient Er^3+^-doped ZYA gain fiber and low-Q cavity, the presented PQS lasers have much higher slope efficiencies than other reported Er^3+^-doped GNR PQS fiber lasers^7^. This efficiency confirms the advantageous optical properties of the GNR thin films. Notably, the average power starts to show roll-over as the pump power reaches 176.4 mW, limiting the power scaling of the PQS fiber lasers. The slightly lower efficiency and higher pump threshold with respect to the mode-locked laser described in the previous section are caused by additional losses from the added thin films. Figure 5c shows the repetition rates and 3-dB pulse durations of the PQS fiber lasers as a function of pump power. The repetition rate increases linearly with the increase in pump power, while the 3-dB pulse duration decreases nonlinearly. The decreased pulse duration is caused by the gain compression^27^. The measured maximum repetition rates of two-layer and three-layer cases are 41.17 kHz to 51.45 kHz, respectively. The recorded minimum 3-dB pulse durations are 5.69 μs and 3.09 μs, which both occur under maximum pump-power conditions. The pulse duration of the three-layer PQS fiber laser is shorter than that of the two-layer laser because of the larger modulation depth. Figure 5d shows the calculated corresponding single-pulse energies and peak powers. The pulse energy of the two-layer PQS fiber laser increases linearly with pump power, but that of the three-layer PQS fiber laser tends to become saturated at pump powers above 182.3 mW. The obtained maximum pulse energy of 403.2 nJ confirms the superior mechanical properties of the GNR thin films for practical applications. Further improvement can be achieved by optimizing the fabrication parameters of the GNR thin-film-based SA. The peak powers of the two PQS fiber lasers are nearly saturated at 70.6 mW (two layer) and 96.48 mW (three layer) because of slight saturation of both the pulse energy and 3-dB pulse duration.

The change from the CW mode-locking regime to the PQS regime can be attributed to the larger modulation depth of the multi-layer GNR SAs. The operation states of a CW mode-locked fiber laser can be determined by the following condition for the intracavity pulse energy *E*_p_^28,29^:

1 $$E_{p}^{2} \ge E_{g } E_{sa } \Delta R,$$

where *E*_P_ is the intracavity pulse energy, *E*_g_ and *E*_sa_ denote the saturation energies of the gain medium and saturable absorber, respectively, and Δ*R* is the modulation depth of the SA. If *E*_p_ does not satisfy Eq. (1), the laser tends to operate in Q-switching or Q-switched mode-locking modes^30^. The single-GNR and multilayer-GNR pulsed fiber lasers have the same *E*_P_ and *E*_sa,_ which are functions of the material characteristics. However, the modulation depth, Δ*R*, increases with the addition of more GNR layers. Thus, the multi-layer GNR SAs have a larger modulation depth, increasing the difficulty of the mode-locked operation for this laser system with respect to the Q-switched operation.

### Summary

In conclusion, this study experimentally demonstrated dual-functional mode-locked and Q-switched Er^3+^-doped YSZA fiber lasers based on GNR-PVA thin films. This is the first study, to the best of our knowledge, in which a colloidal GNR-PVA thin film was used as the SA in a reconfigurable mode-locked and Q-switched fiber laser. High-quality GNR-PVA thin films were fabricated using a wet chemical process. They exhibited a modulation depth of 7.8%, maximum transmission of 93%, and saturation intensity of 6.55 MW/cm^2^. Self-starting mode-locked operation was successfully achieved with an efficiency as high as 24.91% and a maximum pulse energy of 2.3 nJ when a single-layer GNR-PVA thin film was employed. The characteristics of the mode-locked pulses were further verified through theoretical simulations based on a modified nonlinear Schrödinger equation. Q-switching operation was successfully achieved with a maximum average output power of 16 mW and a maximum pulse energy of 0.403 μJ by using multi-layer GNR-PVA thin films to increase the modulation depth of the SA. The GNR -PVA thin films used in these systems were not damaged under several-hour operations. The results verify that the presented colloidal GNR-PVA thin film exhibits superior optical and mechanical properties which make it advantageous for SA applications in dual-functional mode-locked and Q-switched Er^3+^-doped fiber lasers.
